# Progress in Genito-Urinary Surgery

**Published:** 1901-02-16

**Authors:** 


### Progress in Genito-Urinary Surgery.

(*Continued from page* 333.)

**The use of Mercurol in Urethritis.**—Ramon Guiteras,[4] of New York, has published some notes on the use of this remedy. He states that mercurol is a chemical compound of mercury with nucleinic acid—the latter obtained from yeast. It is a brownish-white powder, soluble in water, and does not precipitate albuminous fluids, and is not precipitated by alkalies. The manufacturers claim that the action of the mercury is rendered more effective and less irritating by the combination—so that more mercury may be used without pain or disagreeable symptoms being provoked. As a result of preliminary investigations it was found that the average strength of the solution of mercurol which could be best borne by the patient was grs.x to the ounce, or, approximately, 2 per cent. Guiteras has records of 100 cases treated with the solution of this strength. In 33 of these the gonococcus was examined for, and found in 30 of them. The patients were instructed to inject themselves with the solution after urinating three times a day, and to hold the injection in for five minutes each time. It is pointed out that the action of all germicides is not fully understood. It was supposed that permanganate of potassium injections cured the disease by killing the gonococcus, but later it was found that they gave rise to a sub-mucous œdema which was an unfavourable culture ground for the bacteria. The clinical reports show that of the 100 cases 22 were practically cured in three weeks, 28 in four weeks, 17 in more than four weeks; the remaining cases were still under treatment or had ceased to attend after one or two visits. Guiteras points out that selected cases are more valuable for estimating the value of a drug than a sequence of cases, those being selected in which the treatment was begun early and kept up to the end. He then points out the looseness of the expression "practically cured." A case is definitely cured when the patient is free from symptoms, and has no discharge and no flakes in the urine, and no pus or flakes containing gonococci in the urine after massaging the prostate and seminal vesicles. If the urine is thick after the discharge has ceased the urethritis may be confined to the posterior urethra and flow back into the bladder. Only two of the reported cases suffered from complications, one developing gonorrhœal rheumatism, the other epididymitis, and Guiteras asks "where is there any other solution or mixture that has been employed which does not show a greater percentage of complications. Many writers claim that in 20 per cent. of all cases epididymitis occurs, whereas the rate in these cases was 1 per cent. Another interesting feature was that in only one case was there any marked posterior urethritis. The writer states that it is probable that in from 80 per cent. to 90 per cent. of cases of gonorrhœa the inflammation travels down to the posterior urethra, but that in only 20 per cent. of these are there any acute symptoms due to this, while in this group only 1 per cent. had trouble from that cause. Guiteras concludes that mercurol probably quickly destroys the gonococcus, and lessens the inflammation and tends to prevent complications.

**Tuberculosis of the Testicles and Vesiculæ Seminales.**—Alexis V. Moschowitz writes[5] on the radical treatment of this affection. The course of a case in which a tubercular nodule has appeared in the epididymis is first alluded to. Such a nodule in the course of a few days or weeks will lead to the dissemination of tubercle bacilli through the corresponding vas deferens, which will become infected at first in its scrotal portion, the infection then spreads upwards into the inguinal and abdominal portions, and very soon the seminal vesicle becomes involved. The next step is the infection of the corresponding lobe of the prostate. Thence extension may occur to the posterior urethra and bladder, with secondary infection of the ureters or kidneys. The testicle itself is rarely involved. In some cases the progress of the disease is in the opposite direction, the primary focus being in the seminal vesicle. From these considerations it is obvious that castration will often prove a futile procedure. No operations can be considered radical which do not remove every portion of the diseased tissue, and Moschowitz considers that in the radical operation for this affection the entire vas deferens, with the corresponding seminal vesicle, should be removed together with the testicle, and he recommends a procedure for this purpose which he has carried out in one case. It consists in performing castration as usual, with the exception that the vas deferens is isolated as far up as possible in the inguinal canal by exerting traction, but without running the risk of tearing it. The vas is ligatured and divided at the high level and the proximal end seared with a cautery, and the skin incision closed. The patient is then placed in the lithotomy position with a sound in the bladder, and a curved incision made in front of the anus extending from one tuberosity of the ischium to the other. The space between the rectum and urethra is then dissected up until the prostate and seminal vesicle are reached. With a finger of the left hand in the rectum traction is made on the prostate and vesicle, or the vesicle may be seized in a clamp and gentle traction made. Proceeding in this manner the entire vesicle and the remaining portion of the vas deferens, with the ligature in its free end, can be isolated until the ejaculatory duct is reached, and on the division of that structure the parts come away. The writer then gives summaries of and references to many cases in which the vesiculæ have been removed. The operations have been performed by the inguinal, the sacral, and the perineal route. They enable tubercular nodules to be removed from the prostate. There is a possibility of a urinary fistula arising through the division of the ejaculatory duct.

[4] Lancet, Sept. 1900. [5] Med. Rec. Sept. 1900.

